# *Lactobacillus helveticus* R0052 Ameliorates Cholestasis with FXR-Related Pathways and Modulation of Gut Microbiota

**DOI:** 10.3390/microorganisms14102330

**Published:** 2026-10-13

**Authors:** Xueyang Chen, Li Jiang, Ailing Li, Jian Shen

**Affiliations:** 1 Center for General Practice Medicine, Department of Gastroenterology, Zhejiang Provincial People’s Hospital (Affiliated People’s Hospital), Hangzhou Medical College, No. 158 Shangtang Road, Hangzhou 310014, China; 2 Laboratory of Animal Research Center, The First Affiliated Hospital, Zhejiang University School of Medicine, Hangzhou 310003, China; 3 Shulan (Hangzhou) Hospital, Hangzhou 310003, China

**Keywords:** probiotic, primary sclerosing cholangitis, liver injury, intestinal flora, bile acid

## Abstract

Cholestasis is an important clinical manifestation of primary sclerosing cholangitis (PSC), leading to liver injury, fibrosis and liver failure. The gut microbiota influences the pathogenesis of cholestatic liver disease by modulating metabolic and immune responses. Our study established a PSC mouse model with a 0.1% 3,5-diethoxycarbonyl-1,4-dihydrocollidine (DDC) diet and *Lactobacillus helveticus* R0052 intervention to assess the protective effects of probiotics on the disease. In addition, we investigated the microbiome and transcriptome alterations resulting from *L. helveticus* R0052 intervention to explore associations between gut microbiota and host metabolism. The results showed that *L. helveticus* R0052 reduced liver injury and inflammation, and ameliorated cholestasis in DDC-fed mice. These beneficial effects were accompanied by alterations in hepatic FXR-Cyp7a1 and ileal FXR-Fgf15 signaling pathways, modified bile-acid homeostasis, as well as changes in the gut microbiota and their metabolites. Overall, our research findings provide an experimental basis for the potential application of *L. helveticus* R0052 in the prevention of cholestasis.

## 1. Introduction

Cholestasis is an important clinical manifestation of primary sclerosing cholangitis (PSC) [1], which is characterized by intra- or extrahepatic stenosis with bile duct fibrosis that affects bile circulation and leads to abnormal liver function [2]. From a pathophysiological perspective, biliary inflammation and fibrosis of the hepatic bile ducts can lead to enrichment of bile acids (BAs) [3] and the large amount of hepatic BAs can perpetuate hepatic inflammation, thus leading to subsequent progression to cirrhosis, liver failure, and liver cancer [4]. However, there is still no clinically effective treatment for PSC, and the most definitive treatment remains liver transplantation [5], hence the urgent need to find new effective pharmacological treatments.

The role between the intestinal microecology and the liver is bidirectional, and the pathway consists of a biliary pathway that transfers bile from the liver to the intestine and a portal pathway that transports metabolites from the intestine to the liver [6]. Moreover, the composition of the intestinal flora and the changes in its metabolites play a decisive role in many chronic diseases [7]. The pathogenesis of PSC is still under investigation, but studies have shown that most patients with PSC also have inflammatory bowel disease (IBD) [8,9,10], and many studies have found that intestinal microorganisms affect bile acid homeostasis either directly or indirectly [11,12,13]. *Lactiplantibacillus plantarum* has been shown to alter the enterohepatic circulation of BAs by producing bile salt hydrolase (BSH), which increases Chenodeoxycholic acid (CDCA) levels in the ileum and liver and activates the Farnesoid X receptor (FXR) signaling pathway [14]. Other studies have also shown that *Prevotella copri* can improve cholestasis and liver fibrosis in PSC mouse models by modulating the metabolic and transport pathways of BAs [15]. These studies suggest that probiotics can influence the bile acid cycle in the hepatic-intestinal circulation by modulating the gut microbiota, thereby improving liver inflammation.

*Lactobacillus* has the ability to produce BSH, which catalyzes the conversion of conjugated primary BAs to nonconverted secondary BAs, thereby reducing the reabsorption of BAs in the enterohepatic circulation [16]. *L. helveticus* R0052 has been shown to improve liver diseases in previous studies [17,18]. However, whether *L. helveticus* R0052 can alleviate PSC-related cholestasis and hepatic inflammation remains unclear. Therefore, our study aimed to evaluate the protective effect of *L. helveticus* R0052 on liver inflammation and cholestasis in a 3,5-diethoxycarbonyl-1,4-dihydrocollidine (DDC) feeding-induced PSC mouse model, providing a novel basis for the development of probiotic-based preventive strategies for cholestatic liver disease.

## 2. Materials and Methods

### 2.1. Strain Preparation

The source of probiotic strain *L. helveticus* R0052 was described in the previous article [17]. It was incubated anaerobically in Man Rogosa Sharpe (MRS) broth medium at 37 °C for 24 h. The broth was centrifuged at 4000 rpm for 10 min at 4 °C, washed with PBS and resuspended at 3 × 10^9^ colony-forming units (CFUs)/mL in phosphate-buffered saline (PBS) for gavage.

### 2.2. Animals and Experimental Design

Male C57BL/6J mice (8 weeks old, body weight 22.5 ± 0.5 g) were obtained from Shanghai SLAC Laboratory Animal Co., Ltd. (Shanghai, China). Animals were acclimatized for one week before experiments. Mice were housed at 4 mice per cage under specific pathogen-free (SPF) housing conditions with controlled temperature (22 ± 2 °C), relative humidity (50 ± 10%), and a 12 h light/12 h dark cycle. Environmental enrichment was provided in each cage. Sterile water was available ad libitum. The mice were randomly divided into the normal control (NC) (PBS + control diet) group, the DDC (PBS + DDC diet) group, and the R0052 (*L. helveticus* R0052 + DDC diet) group (*n* = 8 in each group). Mice in the R0052 group were treated with 0.2 mL of *L. helveticus* suspension daily, while the NC and DDC groups were treated with 0.2 mL of PBS by gavage for 14 days. On day 15, the DDC group and R0052 group were fed a 0.1% DDC diet (CAS. D200309, Dyets), while the NC group was supplemented with the control diet for 14 days (Figure S4). Mice were euthanised by injection of sodium pentobarbital on day 28. Death was confirmed by the absence of corneal reflex and cessation of spontaneous respiration for at least 5 min, followed by tissue sample collection. All animal experiments were reviewed by the Ethics Committee (No. 2023-970).

### 2.3. Histopathological Evaluation

Excised liver tissues were fixed in 10% neutral buffered formalin at room temperature for 24 h. After fixation, tissues were processed via routine dehydration, clearing and paraffin embedding to prepare 2 μm sections. Hematoxylin and eosin (H&E) staining of serial sections was used to assess liver inflammation and necrosis, and Sirius Red staining was adopted to quantify hepatic fibrotic changes.

### 2.4. Analysis of Serum Parameters

Serum alanine aminotransferase (ALT), aspartate aminotransferase (AST), alkaline phosphatase (ALP), total bilirubin (TBIL) and direct bilirubin (DBIL) were measured by an automated biochemical analyzer (Hitachi 7600-210; Tokyo, Japan). Serum inflammatory cytokines were measured by the Bio-Plex Pro Mouse Cytokine 23-Plex Assay Kit (Bio-Rad, Hercules, CA, USA).

### 2.5. Liver Bile Acid Composition Analysis

First, approximately 10 mg of liver tissue was weighed, and 20 μL of internal standards [19] dissolved in methanol-water (2:1, *v*/*v*) were added, followed by 980 μL of cold methanol-water solution containing 0.005% HCOOH. Next, the samples were homogenized using a tissue grinder (50 Hz, 90 s) and centrifuged (12,000 rpm, 4 °C, 10 min). The supernatant was collected for ultra-performance liquid chromatography-tandem mass spectrometry (UPLC-MS/MS) detection (Agilent, Santa Clara, CA, USA).

Chromatography was performed on an Agilent ZORBAX Eclipse Plus C18 column (2.1 × 100 mm, 1.8 μm) maintained at 45 °C; injection volume: 1 μL. Mobile phase A: ultrapure water (0.005% formic acid); Mobile phase B: acetonitrile (0.005% formic acid). The gradient-elution program was performed as previously described [20]. MS detection used multiple-reaction monitoring (MRM). Ion-source parameters: drying-gas flow 10 L/min, drying-gas temperature 350 °C, nebulizer pressure 20 psi, sheath-gas temperature 350 °C, sheath-gas flow 10 L/min, capillary voltage 3500 V. Data were acquired and processed by MassHunter B.08.00 (Agilent, USA).

### 2.6. Real-Time PCR Analysis

Total RNA was isolated from liver and ileum tissues using the RNeasy Plus Mini Kit (Qiagen, Hilden, Germany) following the manufacturer’s protocol. Purified RNA was reverse-transcribed into cDNA with PrimeScript RT master mix (TaKaRa Biomedicals, Kusatsu, Japan). Subsequently, mRNA expression was measured with a ViiA7 RT–PCR system (Applied Biosystems, Carlsbad, CA, USA). Each 20 μL qPCR reaction mixture was prepared using SYBR Premix Ex Taq. The thermal-cycling program consisted of an initial denaturation step at 95 °C for 30 s, followed by 40 amplification cycles of 95 °C for 5 s and 60 °C for 30 s. A melting-curve analysis was performed after amplification to verify the specificity of amplified products. Each sample was assayed in two technical replicates. Relative gene expression levels were calculated by the 2^−ΔΔCt^ method using GAPDH as the reference gene. The gene primer sequences used for RT–qPCR analysis are shown in Supplementary Table S1.

### 2.7. 16S rRNA Sequencing

Fecal samples were collected before euthanizing the mice, and DNA was extracted using the DNeasy PowerSoil Pro Kit (Qiagen, Hilden, Germany). PCR amplification of the V3 to V4 variable region of the 16S rRNA gene was then performed using universal primers (343F: 5′-TACGGRAGCAGCAGCAG-3′, 798R: 5′-AAGGTATCTAATCCT-3′). After identification, the data were sequenced on the Illumina NovaSeq6000 platform (Illumina Inc., San Diego, CA, USA), and libraries were constructed.

Qualified raw data were subjected to quality filtering, denoising, read merging and chimera removal using the default parameters of QIIME2 (version 2020.11) to obtain representative sequences and ASV abundance tables. The α-diversity and β-diversity were assessed by QIME2 software. Differences between taxa were also analyzed using linear discriminant analysis (LDA) effect size (LEfSe). The data were uploaded to the SRA database (PRJNA974965).

### 2.8. Metabolome Analysis

Samples were prepared as described previously [21]. Briefly, approximately 30 mg of fecal sample was extracted with methanol-water (1:1, *v*/*v*) containing L-2-chlorophenylalanine as internal standard, followed by grinding, sonication, chloroform addition, and low-temperature centrifugation. The supernatant was dried, then subjected to oximation and silylation derivatization for GC-MS measurement. Chromatographic separation was carried out on a DB-5MS capillary column (30 m × 0.25 mm × 0.25 μm, Agilent J&W Scientific, Santa Clara, CA, USA) with high-purity helium as carrier gas at 1.0 mL/min. The injector temperature was 260 °C; 1 μL sample was injected in split-less mode with a 5 min solvent delay. The oven temperature program started at 60 °C (0.5 min hold), ramped to 125 °C at 8 °C/min, to 210 °C at 8 °C/min, to 270 °C at 15 °C/min, and finally to 305 °C at 20 °C/min (5 min hold).

Mass-spectrometric detection used an electron-impact (EI) ion source at 70 eV; ion-source temperature 230 °C, quadrupole temperature 150 °C. Full-scan mode was applied over *m*/*z* 50–500. Metabolites were annotated by matching mass spectra and retention indices against reference databases, and relative peak abundances were used for subsequent differential-metabolite analysis. The instrument used was GC-MS 7890B-5997A (Agilent, USA).

### 2.9. Liver Transcriptome Analysis

Total RNA was extracted from liver tissue. mRNA was enriched using oligo(dT) magnetic beads, fragmented with NEB Fragmentation Buffer, and used for cDNA synthesis. After end-repair, A-tailing and adapter ligation, 250–300 bp cDNA fragments were PCR-amplified to construct sequencing libraries [22]. Libraries were paired-end-sequenced on the Illumina NovaSeq6000 platform. Clean reads were mapped to the reference genome using HISAT2 (version 2.0.5). Differential-expression analysis was performed by DESeq2, with *p*-values adjusted by the Benjamini–Hochberg method. Genes with *p*-value < 0.05 by DESeq2 were regarded as differentially expressed genes (DEGs). Enrichment analysis of DEGs was performed based on the Kyoto Encyclopedia of Genes and Genomes (KEGG) pathway database. The data were uploaded to the SRA database (PRJNA975522).

### 2.10. Statistical Analysis

Statistical analyses were performed using GraphPad Prism (version 9.4). For three-group comparisons, normality and homogeneity of variance were assessed prior to analysis. One-way ANOVA was applied for normally distributed continuous variables with homogeneous variance, while Brown-Forsythe ANOVA was used for normally distributed data with heterogeneous variance. Significant overall tests were followed by Bonferroni post hoc tests for homogeneous variance data and Dunnett’s T3 post hoc tests for heterogeneous variance data to obtain adjusted *p*-values for multiple-testing correction. The Kruskal–Wallis test was applied for non-normal datasets. The Mann–Whitney U test or Student’s *t* test was used to assess differences between the two groups. The results are expressed as the mean ± standard error of the mean (SEM). *p* < 0.05 was considered significant.

## 3. Results

### 3.1. L. helveticus R0052 Improved Liver Injury in DDC-Fed Mice

The weight in the DDC group was decreased. In contrast, the body weight of mice did not change significantly after *L. helveticus* R0052 intervention (Figure S5).

**Figure 1:**
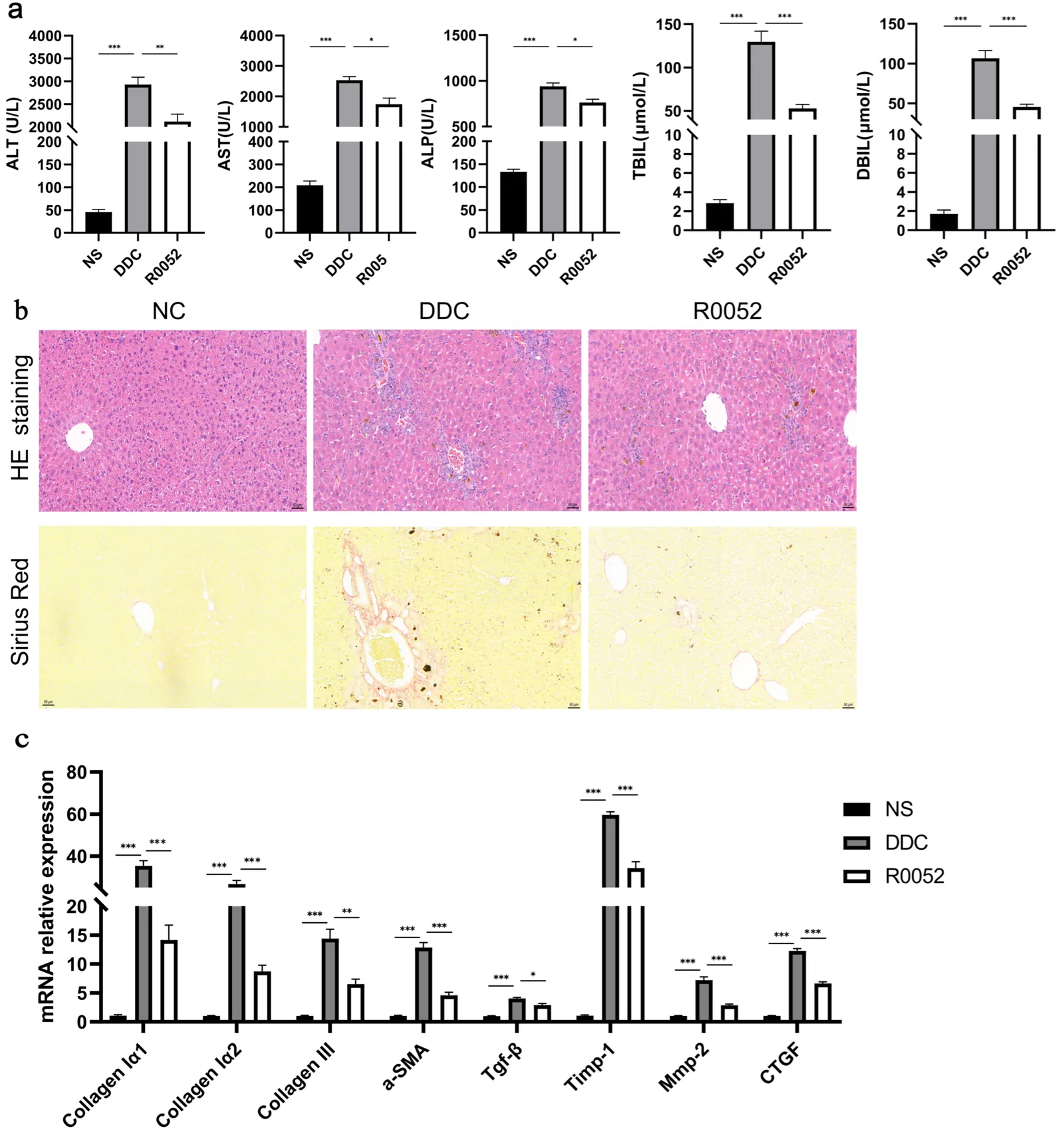
*L. helveticus* R0052 improved liver injury and fibrotic pathology in DDC-fed mice. (**a**) Serum liver function levels. (**b**) Representative pathological sections of the liver stained with H&E and Sirius red (×20). (**c**) The mRNA levels of liver fibrosis genes. Official mouse gene symbols: *Col1a1* (Collagen Iα1), *Col1a2* (Collagen Iα2), *Col3a1* (Collagen III), *Acta2* (α-SMA), *Tgfβ1* (Tgf-β), *Timp1* (Timp-1), *Mmp2* (Mmp-2), *Ctgf* (CTGF). Data are expressed as the mean ± SEM. Compared with the DDC group, * *p* < 0.05, ** *p* < 0.01, *** *p* < 0.001. *n* = 8 per group.

Furthermore, the analysis of liver function tests showed that the liver function of mice in the DDC group was deteriorated, including ALT, AST, ALP, TBIL and DBIL, while the *L. helveticus* R0052 intervention decreased these liver-injury-related biochemical markers (Figure 1a). The results showed that *L. helveticus* R0052 intervention improved liver inflammation in DDC mice.

To assess the hepatic pathological lesions, we stained mouse liver sections with H&E and Sirius Red. Livers from the NC group displayed normal structure. In the DDC group, massive hepatocyte necrosis and prominent peribiliary inflammatory cell infiltration were observed, accompanied by typical onion skin-like fibrotic lesions. In comparison, *L. helveticus* R0052 intervention attenuated DDC-induced hepatocyte necrosis and inflammatory infiltration (Figure 1b).

Consistent with the histological observations, the mRNA expression levels of fibrotic markers, such as Collagen1a1 and α-SMA, were upregulated in the DDC group and partially reduced after *L. helveticus* R0052 intervention (Figure 1c). Overall, the above results indicated that *L. helveticus* R0052 ameliorated DDC-induced liver injury and mitigated fibrotic-related molecular alterations in mice.

### 3.2. L. helveticus R0052 Improved Inflammation Levels (Cytokines) in DDC-Fed Mice

**Figure 2:**
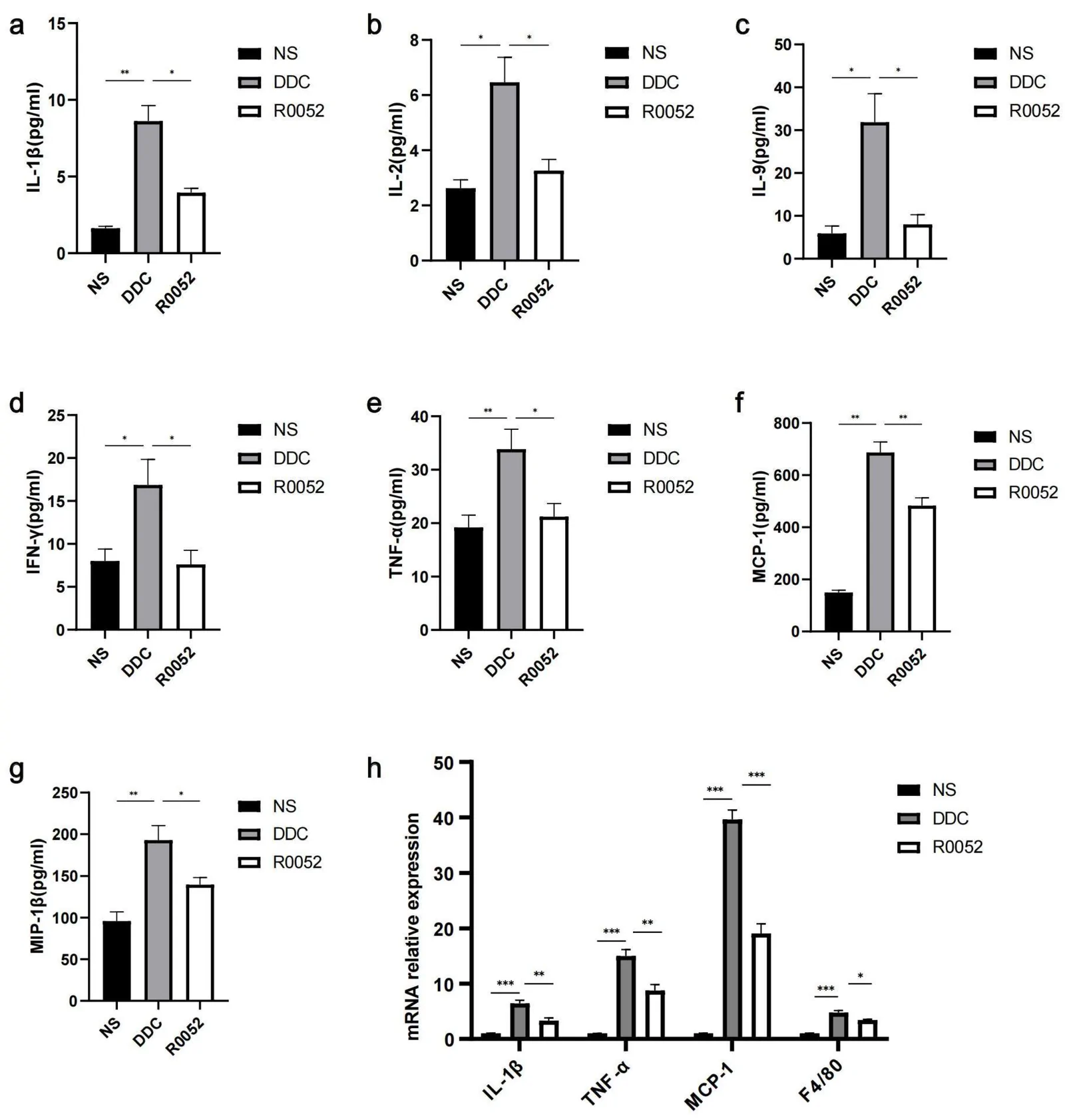
*L. helveticus* R0052 improves inflammation levels in DDC-fed mice. (**a**–**g**) Serum levels of the cytokines IL-1β, IL-2, IL-9, IFN-γ, TNF-α, MCP-1, and MIP-1β. (**h**) The mRNA expression levels of the liver inflammatory factors. Official mouse gene symbols: *Il1b* (IL-1β), *Tnf* (TNF-α), *Ccl2* (MCP-1), *Adgre1* (F4/80). Data are expressed as the mean ± SEM. Compared with the DDC group, * *p* < 0.05, ** *p* < 0.01, *** *p* < 0.001. *n* = 6 per group.

Studies have shown that increased intestinal permeability may affect biliary stasis, which can cause bacterial products to be transferred to the liver and thus worsen the condition [23]. Cytokines are closely related to the progression of inflammation in the disease. To assess the effect of *L. helveticus* R0052 intervention on the inflammatory response, we examined the serum levels of the cytokines in mice and showed that the levels of these cytokines were elevated in the DDC group, while *L. helveticus* intervention decreased the levels of these inflammatory cytokines (Figure 2a–g).

To further assess liver inflammation, we measured the transcription of proinflammatory cytokines and showed that the gene expression of inflammatory markers (IL-1β, TNF-α, MCP-1, and F4/80) was significantly increased in the DDC group and significantly decreased after *L. helveticus* R0052 intervention (Figure 2h).

The serum cytokine levels and liver qPCR results provide strong evidence that *L. helveticus* R0052 protects DDC-fed mice by reducing the production of proinflammatory cytokines in the liver.

### 3.3. L. helveticus R0052 Improves Cholestasis in DDC-Fed Mice

**Figure 3:**
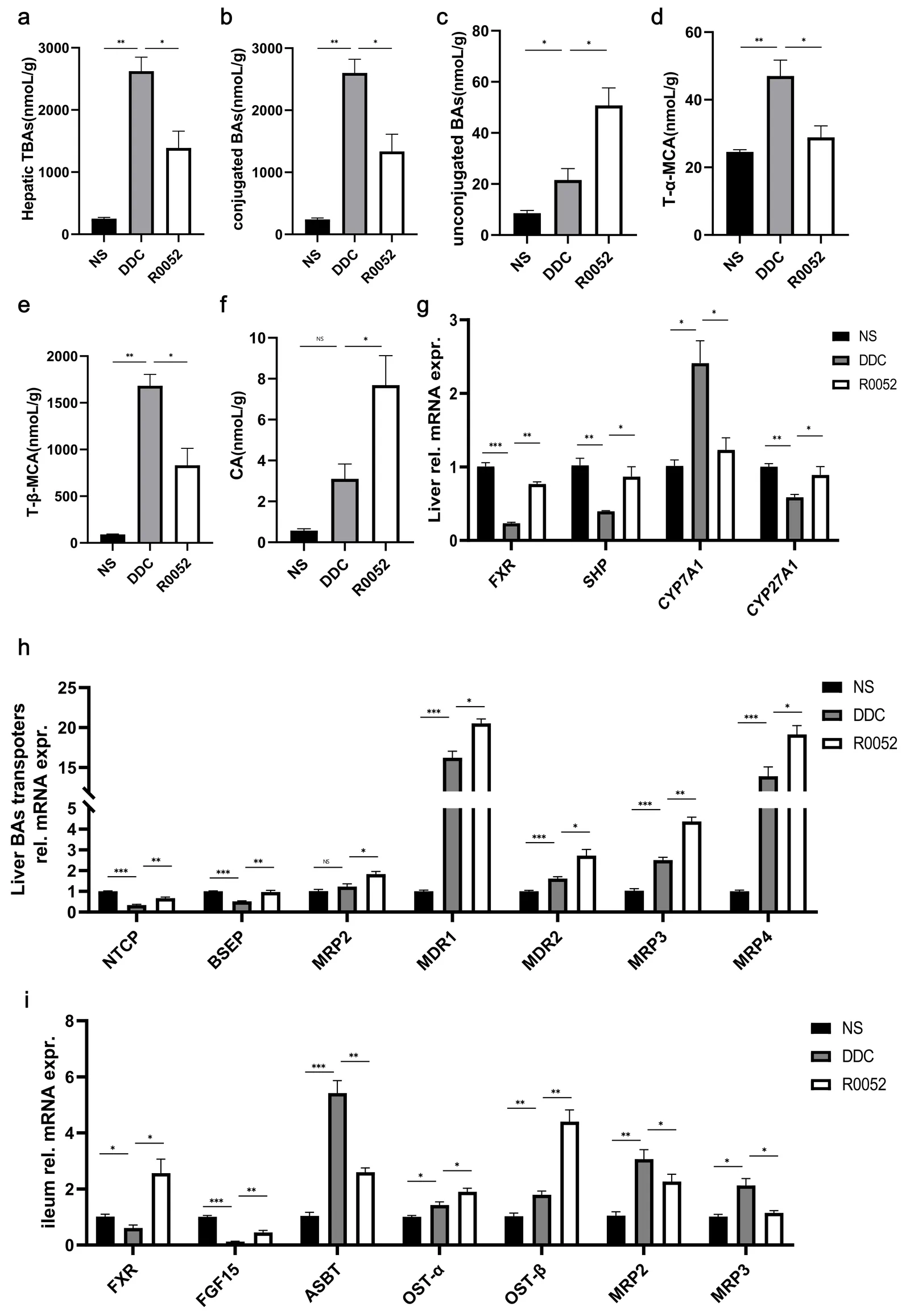
*L. helveticus* R0052 improved bile acid metabolism in DDC-fed mice. (**a**–**f**) Hepatic bile acid levels (*n* = 4 per group). (**g**) The relative expression of FXR, SHP, Cyp7a1 and Cyp27a1 in the liver (*n* = 6 per group). (**h**) The relative expression of hepatic membrane transport proteins (*n* = 6 per group). (**i**) The relative expression of bile-acid-related genes in the ileum (*n* = 6 per group). Official mouse gene symbols: *Nr1h4* (FXR), *Nr0b2* (SHP), *Cyp7a1 *(Cyp7a1), *Cyp27a1 *(Cyp27a1). *Slc10a1* (NTCP), *Abcb11* (BSEP), *Abcc2* (MRP2), *Abcb1a* (MDR1), *Abcb4* (MDR2), *Abcc3* (MRP3), *Abcc4* (MRP4), *Fgf15* (FGF15), *Slc10a2* (ASBT), *Slc51a* (OST-α), *Slc51b* (OST-β). Data are presented as the mean ± SEM. Compared with the DDC group, * *p* < 0.05, ** *p* < 0.01, *** *p* < 0.001. NS indicates no statistically significant difference between groups.

BAs are the main drivers of bile production and secretion, and bile accumulation affects liver inflammation [24]. To investigate whether DDC diet feeding affects the hepatic bile acid profile of mice, we performed targeted quantification of hepatic BAs. The results showed that the DDC group had increased total hepatic BA levels, while the R0052 group had decreased total hepatic BA levels (Figure 3a). We then categorized BAs as conjugated and unconjugated BAs for analysis (Figure 3b,c). Both types of BAs increased in the DDC group, and *L. helveticus* R0052 intervention decreased the level of conjugated BAs but increased the level of unconjugated BAs. Notably, the levels of T-α-MCA, T-β-MCA, and CA were increased in the DDC group, and the *L. helveticus* R0052 intervention decreased the T-α-MCA and T-β-MCA levels but increased the levels of CA (Figure 3d–f).

To explore the molecular mechanisms underlying the protective effects of *L. helveticus* R0052, we detected the expression of key genes involved in hepatic BA metabolism (Figure 3g). The results showed that the mRNA expression of FXR was significantly inhibited in DDC group mice, and this alteration was partially reversed upon *L. helveticus* R0052 intervention. Consistent with the known regulatory cascade, reduced FXR transcript was accompanied by lower small heterodimer partner (SHP) expression in DDC-fed mice, and SHP levels were partially restored following *L. helveticus* R0052 intervention. SHP forms an inhibitory complex with liver receptor homolog-1 (LRH-1), which is a promoter of cholesterol 7α-hydroxylase (Cyp7a1), a key enzyme for bile acid synthesis, and this complex blocks the transcription of Cyp7a1 and SHP itself, thereby inhibiting BA synthesis [25]. As our results showed, the Cyp7a1 mRNA expression level in the liver was significantly increased in the DDC group, but the expression level of cholesterol 27-hydroxylase (Cyp27a1) was slightly decreased compared to that in the NC group, while *L. helveticus* R0052 intervention decreased the expression level of Cyp7a1 and increased the expression level of Cyp27a1. These gene expression changes were concomitant with altered hepatic BA homeostasis in DDC mice following *L. helveticus* R0052 intervention.

On the other hand, in addition to reducing BA synthesis, multiple membrane transport proteins in the liver play key roles in maintaining BA homeostasis (Figure 3h). Compared to those in the NC group, the mRNA expression of the Na^+^-taurocholate cotransporting polypeptide (NTCP) was significantly suppressed, and the bile salt export pump (BSEP) was reduced; the levels of multidrug resistance-associated protein (MRP2) were comparable, and the mRNA expression of multidrug resistance protein (MDR1), MDR2, MRP3 and MRP4 was upregulated in the DDC group. However, compared to the DDC group, *L. helveticus* R0052 intervention reversed the mRNA levels of NTCP and BSEP and further increased the mRNA expression of the efflux transport proteins MDR1, MDR2, MRP3 and MRP4.

We also performed qPCR analysis on ileum tissue (Figure 3i), and our results indicated that lower FXR mRNA expression was observed in DDC-fed mice. Similarly, Fibroblast growth factor 15 (FGF15) mRNA expression in DDC-fed mice was substantially reduced, but *L. helveticus* R0052 intervention reversed the expression of FXR and FGF15. The mRNA expression of apical sodium-dependent bile acid transporter (ASBT), Mrp2, Mrp3, organic solute transporter (OST)-α, and OST-β increased in the DDC group. However, compared to the DDC group, the mRNA expression of ASBT, Mrp2 and Mrp3 was reversed in the R0052 group, while the mRNA expression levels of OST-α and OST-β were higher. These transcriptional alterations in the ileum were correlated with changes in the intestinal FXR-FGF15-related gene signature and hepatic BA metabolic profiles.

### 3.4. L. helveticus R0052 Restores the Intestinal Flora in DDC-Fed Mice

**Figure 4:**
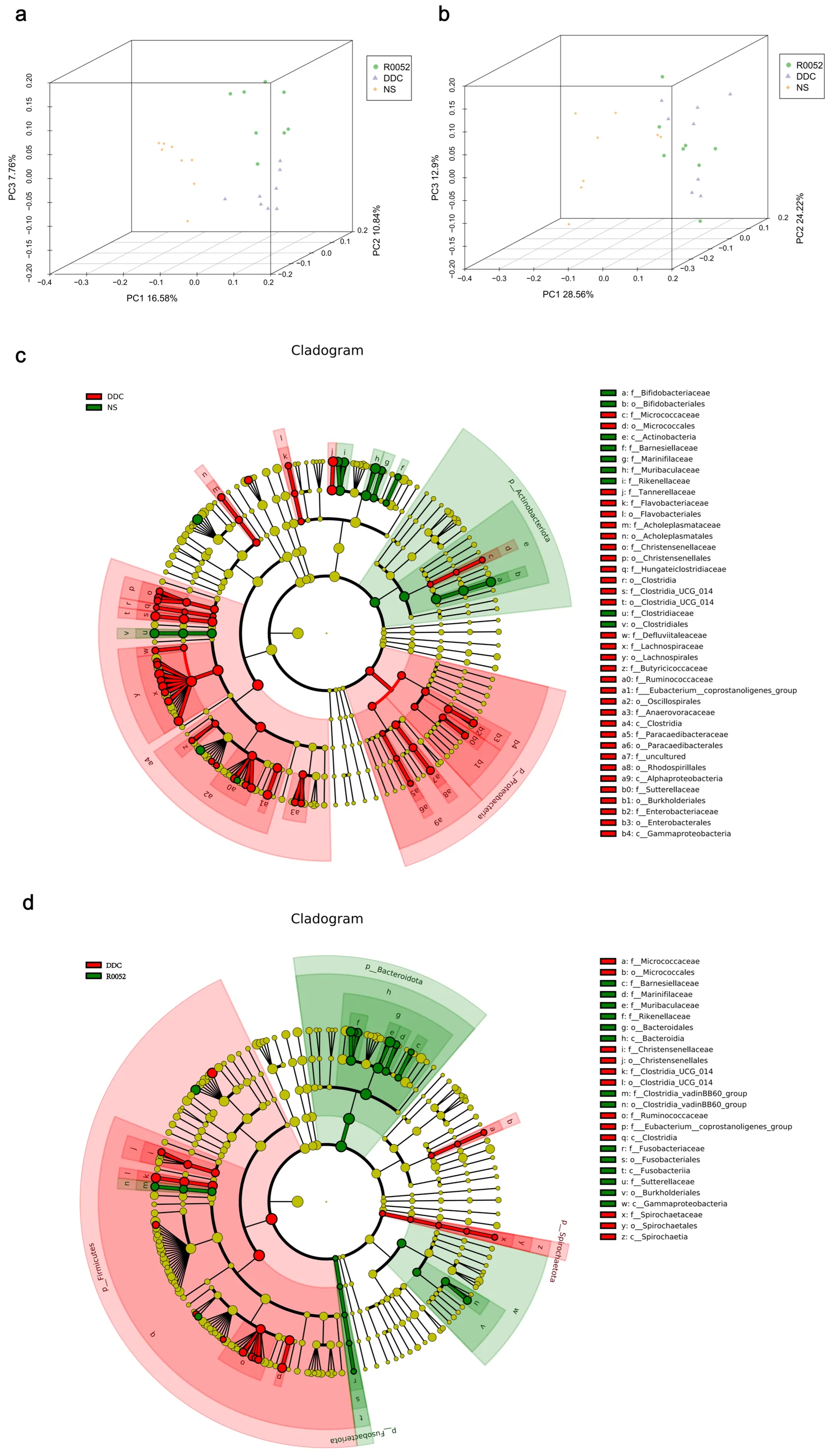
The effects of *L. helveticus* R0052 on the alterations in the gut microbiota in DDC-fed mice. Principal coordinate analysis (PCoA) plot with unweighted UniFrac distances (**a**) and weighted UniFrac distances (**b**). The relative abundances of bacterial taxa differed between the NC and DDC groups (**c**) and the DDC and R0052 groups (**d**). *n* = 8 per group.

We performed 16S rRNA analysis on collected fecal samples to explore the effect of *L. helveticus* on the gut microbiome. The α-diversity analysis, as evaluated by the ACE and Simpson indices, showed no significant differences in microbial richness and diversity among the three groups (Figure S6). For β-diversity, principal coordinate analysis (PCoA) based on unweighted and weighted UniFrac distances revealed clear separation of microbial community profiles across the three groups. Consistently, PERMANOVA analysis further confirmed significant differences in gut microbiota structure among groups (*p* = 0.001 for both unweighted and weighted UniFrac distances; Figure 4a,b).

To assess the improvement in microbial function by *L. helveticus* R0052, we performed a linear discriminant analysis effect size (LEfSe) pathway analysis on the intestinal flora structure to screen the characteristic biomarkers in each group. Compared to the NC group (Figure 4c and Figure S1a), the DDC group was enriched with *Micrococcaceae*, *Tannerellaceae*, *Flavobacteriaceae*, *Acholeplasmataceae*, *Christensenellaceae*, *Hungateiclostridiaceae*, *Clostridia_UCG_014*, *Defluviitaleaceae*, *Lachnospiraceae*, *Butyricicoccaceae*, *Ruminococcaceae*, *Eubacterium*, *Anaerovoracaceae*, *Paracaedibacteraceae*, *Sutterellaceae*, and *Enterobacteriaceae*. *Bifidobacteriaceae*, *Barnesiellaceae*, *Marinifilaceae*, *Muribaculaceae*, *Rikenellaceae*, *Clostridiaceae* and *Clostridiales* were depleted in the DDC group.

Compared to the R0052 group (Figure 4d and Figure S1b), the DDC group was enriched with *Micrococcaceae*, *Christensenellaceae*, *Clostridia_UCG_014*, *Ruminococcaceae*, *Eubacterium* and *Spirochaetaceae*. The DDC group was depleted of *Bacteroidales*, *Barnesiellaceae*, *Marinifilaceae*, *Muribaculaceae*, *Rikenellaceae*, *Clostridia*, *Fusobacteriaceae* and *Sutterellaceae*.

### 3.5. L. helveticus R0052 Restored Metabolic Disorders in DDC-Fed Mice

**Figure 5:**
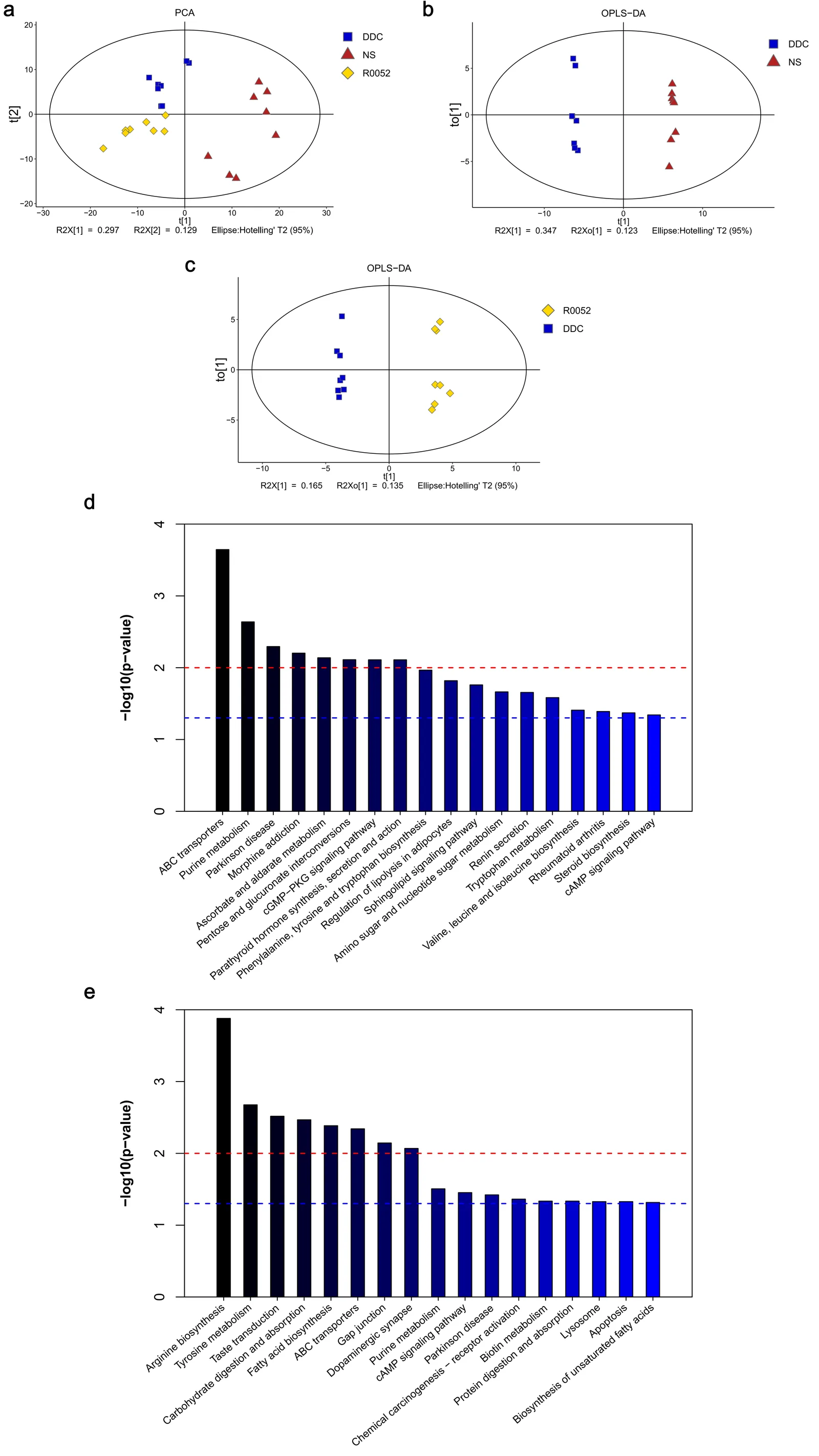
*L. helveticus* R0052 alleviates DDC-induced intestinal metabolism disorders by GC-MS analysis. (**a**) PCA plot illustrating clear separation of metabolic profiles. OPLS-DA plot illustrating separation of gut metabolic profiles of the NC group and DDC group (**b**) and the DDC group and R0052 group (**c**). (**d**) KEGG enrichment analysis between the NC group and DDC group. (**e**) KEGG enrichment analysis between the DDC group and R0052 group. *n* = 8 per group. Note: Enriched KEGG terms represent statistical enrichment of metabolite annotations obtained from intestinal GC-MS data; these findings do not equate to functional activation or inhibition of host intracellular signaling pathways. The y-axis indicates −log_10_(*p*-value). The red dashed line corresponds to *p* = 0.01, and the blue dashed line corresponds to *p* = 0.05, which are the significance thresholds for KEGG pathway enrichment.

To further explore the amelioration mechanism of *L. helveticus* R0052 in DDC-fed mice, the GC–MS analyses of intestinal metabolites were performed. GC–MS analysis also showed that each group was clearly separated in the PCA plot (Figure 5a) and the OPLS-DA plot (Figure 5b,c). The heatmap showed an increase in the levels of fatty acids such as tridecylic acid, 3-aminoglutaric acid and sebacic acid in the DDC group and a decrease in fatty acids such as 2-undecenoic acid in the NC group. In addition, the levels of fatty acids such as myristic acid, palmitic acid, petroselinic acid, oleic acid and palmitelaidic acid were increased in the R0052 group, while fatty acids such as 17-octadecynoic acid and nonadecylic acid were decreased in the DDC group (Figures S2 and S3).

The pathways enriched between the DDC group and the NC group included purine metabolism; pentose and glucuronate interconversions; cGMP-PKG signaling pathway; phenylalanine, tyrosine and tryptophan biosynthesis; regulation of lipolysis in adipocytes; sphingolipid signaling pathway; amino sugar and nucleotide sugar metabolism; tryptophan metabolism; valine, leucine and isoleucine biosynthesis; and cAMP signaling pathway (Figure 5d).

The pathways enriched between the DDC and R0052 groups included arginine biosynthesis, tyrosine metabolism, carbohydrate digestion and absorption, fatty acid biosynthesis, purine metabolism, cAMP signaling pathway, biotin metabolism, protein digestion and absorption, lysosome, apoptosis and biosynthesis of unsaturated fatty acids (Figure 5d).

### 3.6. L. helveticus R0052 Improves Hepatic Transcription in DDC-Fed Mice

**Figure 6:**
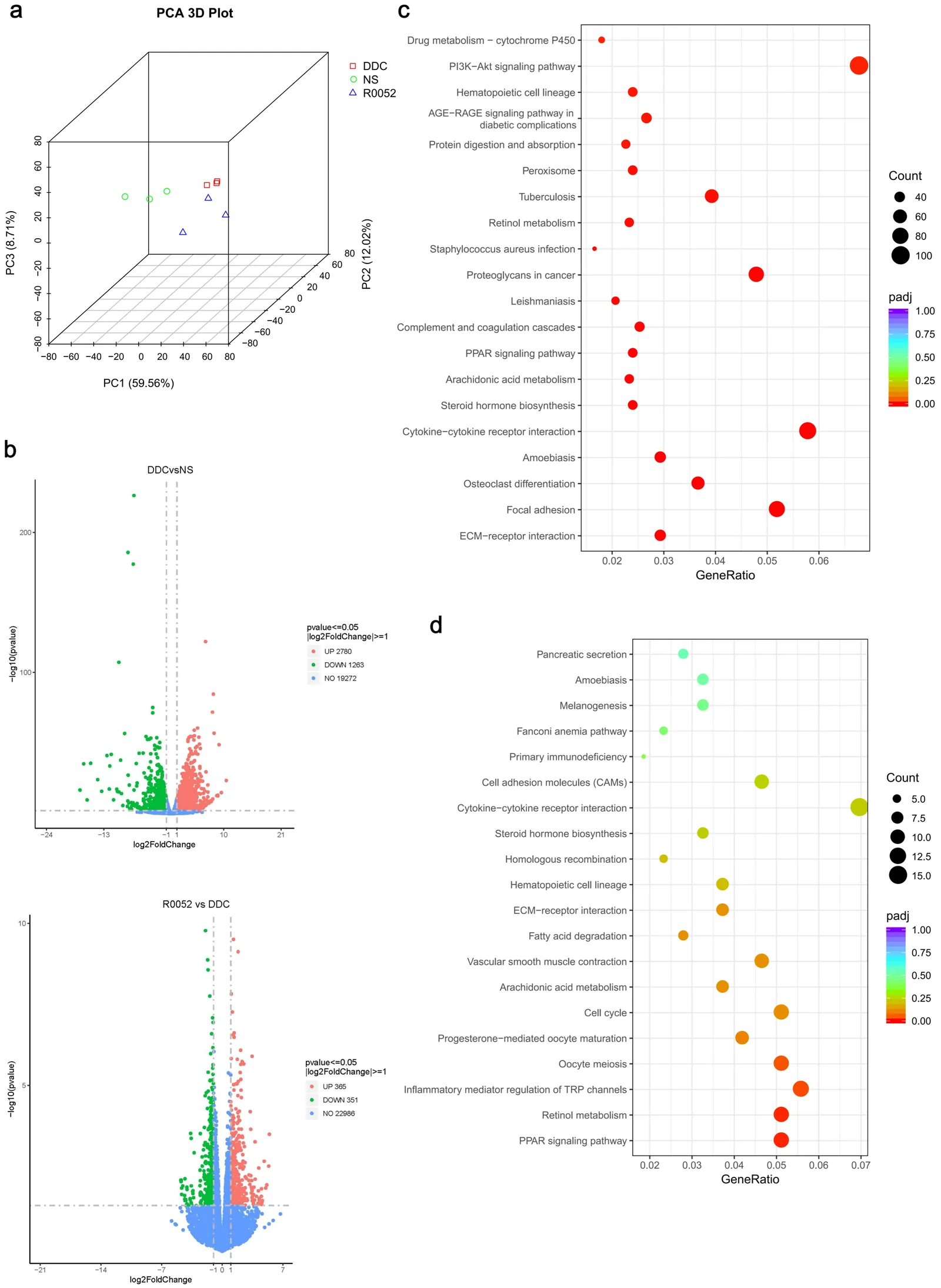
The liver transcriptome was altered by DDC and alleviated by *L. helveticus* R0052 in the liver. (**a**) PCA plot illustrating the transcriptome profiles of the three groups. (**b**) Volcano plot results of the differentially expressed genes in the different groups. KEGG pathway enrichment analysis of the NC and DDC groups (**c**) and the DDC and R0052 groups (**d**). *n* = 3 per group.

Cholestasis affects fatty acid metabolism in the liver [26]. To demonstrate that *L. helveticus* R0052 ameliorates the possible target genes of cholestasis, RNAseq analysis was used to assess the hepatic gene expression profile obtained. According to the PCA results (Figure 6a), separation was observed among the 3 groups, with the DDC group separated from both the NC group and the R0052 group, indicating that the NC group was more similar to the R0052 group than the DDC group. To better understand the regulation of *L. helveticus* R0052 in gene expression profiles, differentially expressed genes (DEGs) were identified, with a total of 4043 differentially expressed genes between the DDC group and NC group and 716 genes between the R0052 group and the DDC group. Volcano plots showed that 2780 DEGs were significantly upregulated and 1263 DEGs were downregulated between the DDC group and the NC group (Figure 6b), with the differentially expressed genes involved in regulating inflammatory processes, bile acid and lipid metabolism, and other compound metabolic processes. Volcano plots revealed 365 DEGs that were significantly upregulated and 351 DEGs that were downregulated between the R0052 group and the DDC group (Figure 6b).

KEGG enrichment analysis was performed on the DEGs, and the enriched pathways between the DDC group and NC group included ECM-receptor interaction, PPAR signaling pathway, cytokine–cytokine receptor interaction, steroid hormone biosynthesis, complement and coagulation cascades, arachidonic acid metabolism, retinol metabolism, protein digestion and absorption, and PI3K−Akt signaling pathway (Figure 6c). The differentially expressed gene enrichment pathways between the DDC and R0052 groups included the PPAR signaling pathway, retinol signaling pathway, inflammatory mediator regulation of TRP channels, regulation of TRP channels, retinol metabolism, and oocyte meiosis (Figure 6d).

## 4. Discussion

PSC is a chronic liver disease whose clinical manifestations are dominated by liver inflammation and bile duct stenosis due to fibrosis [2,8]. At this stage, there is no effective pharmacological treatment. *L. helveticus* R0052, as a potential probiotic for liver disease treatment [18,27,28], has not been explored for its effect on sclerosing cholangitis. In our study, we found that feeding the DDC diet led to significant liver inflammation, fibrosis and cholestasis in mice, while intervention with *L. helveticus* R0052 reversed the adverse effects of the diet on DDC-fed mice, thereby reducing liver injury and cholestasis.

Serum biochemical parameters are sensitive indicators of liver injury. Our results showed that administration of *L. helveticus* R0052 significantly ameliorated DDC-induced liver injury. Many molecules are involved in each phase of the preimmune and late inflammatory response, with TNF-α being regulated by NF-κB [29]. In particular, proinflammatory cytokines such as TNF-α may reduce bile flow and thus aggravate cholestasis [30], the development of which can lead to further liver inflammation and fibrosis.

We also found that acute cholestasis induces altered gene expression that is involved in fatty acid and amino acid metabolism. Cholestasis induces changes in many genes, including extracellular matrix, autophagy, inflammation and lipid metabolism. Severe liver injury with acute cholestasis was induced in DDC-fed mice, and the protective effect of *L. helveticus* R0052 was associated with different hepatic gene expression profiles, mainly related to tissue repair, metabolism and immune function.

Cholesterol in the liver is converted by cytochrome P450 to form bile acids, which later form primary bile acids through two pathways [31], which are bound to glycine and taurine and secreted into the intestine [32]. Whereas previous studies have suggested that the accumulation of BAs in the liver promotes cellular damage [33], the results targeting bile acids in our liver analyses showed that the TBA levels and bound bile acid levels were significantly elevated in the DDC group and were significantly improved after *L. helveticus* R0052 intervention, indicating that the intervention ameliorated DDC-induced hepatic cholestatic metabolic disorders. T-α-MCA and T-β-MCA are natural FXR antagonists [12], and nonconjugated bile acids (e.g., CA) can act as important agonists of FXR [34], while our results showed that the DDC group increased the levels of T-α-MCA and T-β-MCA, but *L. helveticus* R0052 intervention decreased the levels of both and increased the levels of CA, suggesting that intervention with *L. helveticus* R0052 may increase the expression of FXR in DDC-fed mice. In addition, FXR plays a large role in the regulation of hepatic bile acid biosynthesis, transport and secretion [35]. Our results also suggested that the levels of FXR were suppressed in the DDC group of mice, and *L. helveticus* R0052 intervention upregulated the mRNA expression of FXR in the liver. Activated FXR not only induced elevated SHP to inhibit the expression of Cyp7a1, the rate-limiting enzyme of bile acid synthesis [36,37], but also induced transport proteins (BSEP, MDR2 and MRP2) to promote bile secretion and reduce bile acid accumulation by enhancing basolateral efflux pumps (MRP3 and MRP4) [38]. In contrast, in the ileum, previous studies have shown that BA synthesis in the liver is regulated by the FXR-FGF15/19 axis [39], which is consistent with our findings. Our results showed that FXR-associated gene expression was altered in the ileum after *L. helveticus* R0052 intervention, accompanied by increased FGF15 and decreased ASBT. These molecular alterations may reduce the reabsorption of conjugated BAs and thereby contribute to the suppression of hepatic BA synthesis [40]. OST-α, OST-β, MRP2 and MRP3 are important basolateral bile acid transport proteins in the ileum [34] that excrete BAs into the portal circulation and are important alternative efflux pathways [41]. In our study, we observed elevated mRNA expression of these transporters in both DDC and R0052 groups, and these transporters have been reported to contribute to lowering hepatic bile-salt burden in earlier work [42]. In conclusion, our study suggests that *L. helveticus* R0052 ameliorated disrupted BA distribution and metabolism in the enterohepatic circulation. Two sets of transcript-level changes were observed following intervention. On the one hand, FXR-associated gene transcripts in the liver and ileum were markedly changed, accompanied by shifts in hepatic SHP and Cyp7a1, as well as ileal FGF15 mRNA levels. These transcriptional changes may correlate with suppressed hepatic BA synthesis. On the other hand, multiple BA excretory transporters exhibited elevated mRNA expression in the liver and ileum of *L. helveticus* R0052-treated mice. Collectively, these transcript-level alterations may be associated with rebalanced BAs in the enterohepatic circulation.

BAs can affect the host gut microbiota by disrupting bacterial membranes, denaturing their proteins and causing oxidative damage to DNA [26]. Any alteration in either organ may affect the BA pool and thus the overall homeostasis of the host [43]; thus, gut microbiota exerts critical regulatory effects on bile acid metabolism and enterohepatic recirculation. Primary BAs are converted to secondary BAs during transit, and the gut microbiota has been shown to play a key role in the biotransformation of BAs through the processes of debonding, dehydroxylation and rebonding [44]. The gut flora is closely associated with disease, and the intestinal flora plays an important role in BA synthesis. Our results showed that the abundances of Bacteroidales and Muribaculaceae were increased in the R0052 group compared to the DDC group, and previous studies have shown that Muribaculaceae can regulate the T-α-MCA contents and can increase the expression of MDR1 [15], while the abundance of Bacteroidales correlated with fecal DCA levels [45], suggesting that differences in gut flora may influence the process of BA cycling and modulate liver damage, providing a rationale for new therapies based on regulation of the gut microbiota.

The intestinal microbiota is involved in metabolic processes that affect intestinal barrier function, the immune system and host gut homeostasis, including short-chain fatty acids and trimethylamine-N-oxide [46]. Probiotics can remodel host fatty acid metabolism and alter circulating fatty acid profiles, as reported in previous studies investigating probiotic-derived metabolites in fermented food systems and the metabolic characteristics of lactic acid bacteria [47,48]. Consistently, previous studies have demonstrated that the gut microbiota maintains its regulatory role in lipid metabolism by promoting the desaturation and elongation of fatty acids [49]. Our results showed that *L. helveticus* R0052 was associated with DDC-induced alterations in intestinal metabolites, including altered fatty acid profiles.

Several limitations of the present study should be acknowledged. The DDC-fed mouse model only partially recapitulates features of human PSC. The intervention adopted a preventive rather than therapeutic setting, and a dedicated probiotic-only control group under physiological conditions was not included. Importantly, we did not assess strain-specific intestinal colonization of *L. helveticus* R0052, leaving open whether the observed effects require stable gut colonization. Meanwhile, we observed concurrent changes in gut microbiota, bile acid metabolism and hepatic phenotypes, yet this study cannot confirm that microbiota alterations drive the observed hepatic effects; further experimental validation is therefore required. Our work is also limited by the absence of independent replication and human sample validation. In future studies, we will continue to elucidate the mechanism underlying bile-acid-modifying effects of *L. helveticus* R0052 and lay a foundation for subsequent translational research.

## 5. Conclusions

Overall, the probiotic *L. helveticus* R0052 intervention alleviated DDC-induced cholestasis and hepatic inflammation, with modified bile acid homeostasis in mice. These improvements were associated with reshaped gut microbiota profiles linked to cholestatic liver injury phenotypes. Therefore, probiotics targeting gut microbiota to modulate bile acid homeostasis represent a promising strategy for mitigating biliary inflammation and liver injury in PSC.

## Data Availability

Raw 16S rRNA and liver transcriptomic sequencing data have been deposited in NCBI Sequence Read Archive (SRA) under BioProject PRJNA974965 and PRJNA975522.

## Supplementary Materials

The following supporting information can be downloaded at: https://www.mdpi.com/article/10.3390/microorganisms14102330/s1, Figure S1. The LEfSe branching plot of gut microbiota in three groups mice. (a) LEfSe branching plot between the NC group and DDC group. (b) LEfSe branching plot between the DDC group and R0052 group; Figure S2. Heatmap of the different metabolites between the NC group and DDC group; Figure S3. Heatmap of the different metabolites between the DDC group and R0052 group; Figure S4. Experimental flowchart; Figure S5. Daily mice body weight changes in NC, DDC and R0052 groups; Figure S6. Alpha-diversity evaluation based on Chao1 and Simpson indexes; Table S1. Primer sequences used for Real-time PCR studies on mice. Table S2. Multiplicity-adjusted *p*-values obtained from post-hoc pairwise comparisons.
