# Supplementary material for: Lactobacillus helveticus R0052 Ameliorates Cholestasis with FXR-Related Pathways and Modulation of Gut Microbiota
Source: Microorganisms. 2026 Oct 13;14(10):2330. doi: 10.3390/microorganisms14102330 (PMC13708005; doi:10.3390/microorganisms14102330)
Supplement: Supplementary file 1 [file microorganisms-14-02330-s001.zip › microorganisms-4563934-supplementary.pdf]

Supplemental Material

Table S1: Primer sequences used for Real-time PCR studies on mice.

	Gene	Forward Sequence (5'-3')	Reverse Sequence (5'-3')
Gapdh	**GAPDH**	TGCGACTTCAACAGCAACTC	ATGTAGGCAATGAGGTCCAC
Col1a1	**Collagen 1α1**	CTGGCGGTTCAGGTCCAAT	TTCCAGGCAATCCACGAGC
Col1a2	**Collagen 1α2**	GGAGAGAAGGGAACCAAAGG	AGGGAAGCCAGTCATACCAG
Col3a1	**Collagen III**	CTGGTCAGCCTGGAGATAAG	ACCAGGACTACCACGTTCAC
Acta2	**a-SMA**	AATGGCTCTGGGCTCTGTAA	CTCTTGCTCTGGGCTTCATC
Tgfβ1	**Tgf-β**	CCACCTGCAAGACCATCGAC	CTGGCGAGCCTTAGTTTGGAC
Timp1	**Timp-1**	GCATCTCTGGCATCTGGCATC	GGTATAAGGTGGTCTCGTTGA
Mmp2	**Mmp-2**	GCTGATACTGACACTGGTACTG	CAATCTTTTCTGGGAGCTC
Ctgf	**CTGF**	GGGCCTCTTCTGCGATTTC	ATCCAGGCAAGTGCATTGGTA
Il1b	**IL-1β**	GAAATGCCACCTTTTGACAGTG	TGGATGCTCTCATCAGGACAG
Tnf	**TNF-α**	TGTCCCTTTCACTCACTGGC	CATCTTTTGGGGGAGTGCCT
Ccl2	**MCP-1**	GGCTGGAGAGCTACAAGAGG	ATGTCTGGACCCATTCCTTC
Adgre1	**F4/80**	TGACTCACCTTGTGGTCCTAA	CTTCCCAGAATCCAGTCTTTCC
Nr1h4	**FXR**	CTTGATGTGCTACAAAAGCTGTG	ACTCTCCAAGACATCAGCATCTC
Nr0b2	**SHP**	TCTGCAGGTCGTCCGACTAT	CAGGCAGTGGCTGTGAGAT
Cyp7a1	**Cyp7a1**	AACAACCTGCCAGTACTAGATAGC	GTGTAGAGTGAAGTCCTCCTTAGC
Cyp27a1	**Cyp27a1**	CCAGGCACAGGAGAGTACG	GGGCAAGTGCAGCACATAG
Slc10a1	**NTCP**	TCATCTGCGGCTGCTCTCC	TGGTCATCACAATGCTGAGGTTC
Abcb11	**BSEP**	GCTGCCAAGGATGCTAATGC	CTACCCTTTGCTTCTGCCCA
Abcc2	**MRP2**	GCTTCCCATGGTGATCTCTT	ATCATCGCTTCCCAGGTACT
Abcb1a	**MDR1**	GAAATGCGATGAAGAAAGCA	CGGAAACAAGCAGCATAAGA
Abcb4	**MDR2**	ATCCTATGCACTGGCCTTCTGGT	GAAAGCATCAATACAGGGGGCAG
Abcc3	**MRP3**	TGAGATCGTCATTGATGGGC	AGCTGAGAGCGCAGGTCG

Abcc4	**MRP4**	TTAGATGGGCCTCTGGTTCT	GCCCACAATTCCAACCTTT
Fgf15	**FGF15**	GAGGACCAAAACGAACGAAATT	ACGTCCTTGATGGCAATCG
Slc10a2	**ASBT**	ACCACTTGCTCCACACTGCTT	CGTTCCTGAGTCAACCCACAT
Slc51a	**OST-α**	TGTTCCAGGTGCTTGTCATCC	CCACTGTTAGCCAAGATGGAGAA
Slc51b	**OST-β**	GATGCGGCTCCTTGGAATTA	GGAGGAACATGCTTGTCATGAC

Table S2: Multiplicity-adjusted P-values obtained from post-hoc pairwise comparisons.

Measured parameter	Adjusted p-value (DDC vs NC)	Adjusted p-value (R0052 vs DDC)
**ALT**	$p < 0.0001$	0.0099
**AST**	$p < 0.0001$	0.0162
**ALP**	$p < 0.0001$	0.0145
**TBIL**	$p < 0.0001$	0.0007
**DBIL**	$p < 0.0001$	0.0007
**IL-1β**	0.0028	0.0124
**IL-2**	0.0195	0.0409
**IL-9**	0.0248	0.0388
**IFN-γ**	0.0296	0.0227
**TNF-α**	0.0087	0.0233
**MCP-1**	0.0001	0.0085
**MIP-1β**	0.0002	0.0285
**hepatic BAs**	0.0044	0.0341
**conjugated BAs**	0.0042	0.0312
**unconjugated BAs**	0.0482	0.0487
**T-α-MCA**	0.0023	0.0304
**T-β-MCA**	0.0029	0.0105
**CA**	0.1725	0.0171
***Gapdh*(GAPDH)**	-	-
***Col1a1*（Collagen 1α1）**	p < 0.0001	0.0003
***Col1a2*（Collagen 1α2）**	p < 0.0001	p < 0.0001
***Col3a1*（Collagen III）**	0.0008	0.0055
***Acta2*（a-SMA）**	p < 0.0001	p < 0.0001
***Tgfβ1*（Tgf-β）**	p < 0.0001	0.0268
***Timp1*（Tgf-β）**	p < 0.0001	0.0002

Mmp2（**Mmp-2**）	0.0002	0.0006
Ctgf（**CTGF**）	$p < 0.0001$	$p < 0.0001$
Il1b（**IL-1β**）	0.0004	0.0044
Tnf（**TNF-α**）	0.0001	0.0053
Ccl2（**MCP-1**）	$p < 0.0001$	$p < 0.0001$
Adgre1（**F4/80**）	0.0003	0.0286
Nr1h4（**FXR, liver**）	$p < 0.0001$	$p < 0.0001$
Nr0b2（**SHP**）	0.0024	0.0326
Cyp7a1（**Cyp7a1**）	0.0101	0.0197
Cyp27a1（**Cyp27a1**）	$p < 0.0001$	0.0397
Slc10a1（**NTCP**）	$p < 0.0001$	0.0010
Abcb11（**BSEP**）	$p < 0.0001$	0.0023
Abcc2（**MRP2**）	0.4049	0.0188
Abcb1a（**MDR1**）	$p < 0.0001$	0.0036
Abcb4（**MDR2, liver**）	0.0006	0.0261
Abcc3（**MRP3, liver**）	$p < 0.0001$	0.0001
Abcc4（**MRP4**）	0.0002	0.0161
Nr1h4（**FXR, ileum**）	0.0235	0.0207
Fgf15（**FGF15**）	$p < 0.0001$	0.0096
Slc10a2（**ASBT**）	0.0002	0.0018
Slc51a（**OST-α**）	0.0217	0.0395
Slc51b（**OST-β**）	0.0031	0.0021

Abcb4 **(MDR2, ileum)**	0.0021	0.0484
Abcc3 **(MRP3, ileum)**	0.0103	0.0187

Footnote: Adjusted P-values were derived from Bonferroni post-hoc tests (for data with homogeneous variance) or Dunnett’s T3 post-hoc tests (for data with heterogeneous variance) following one-way ANOVA or Brown-Forsythe ANOVA, respectively.

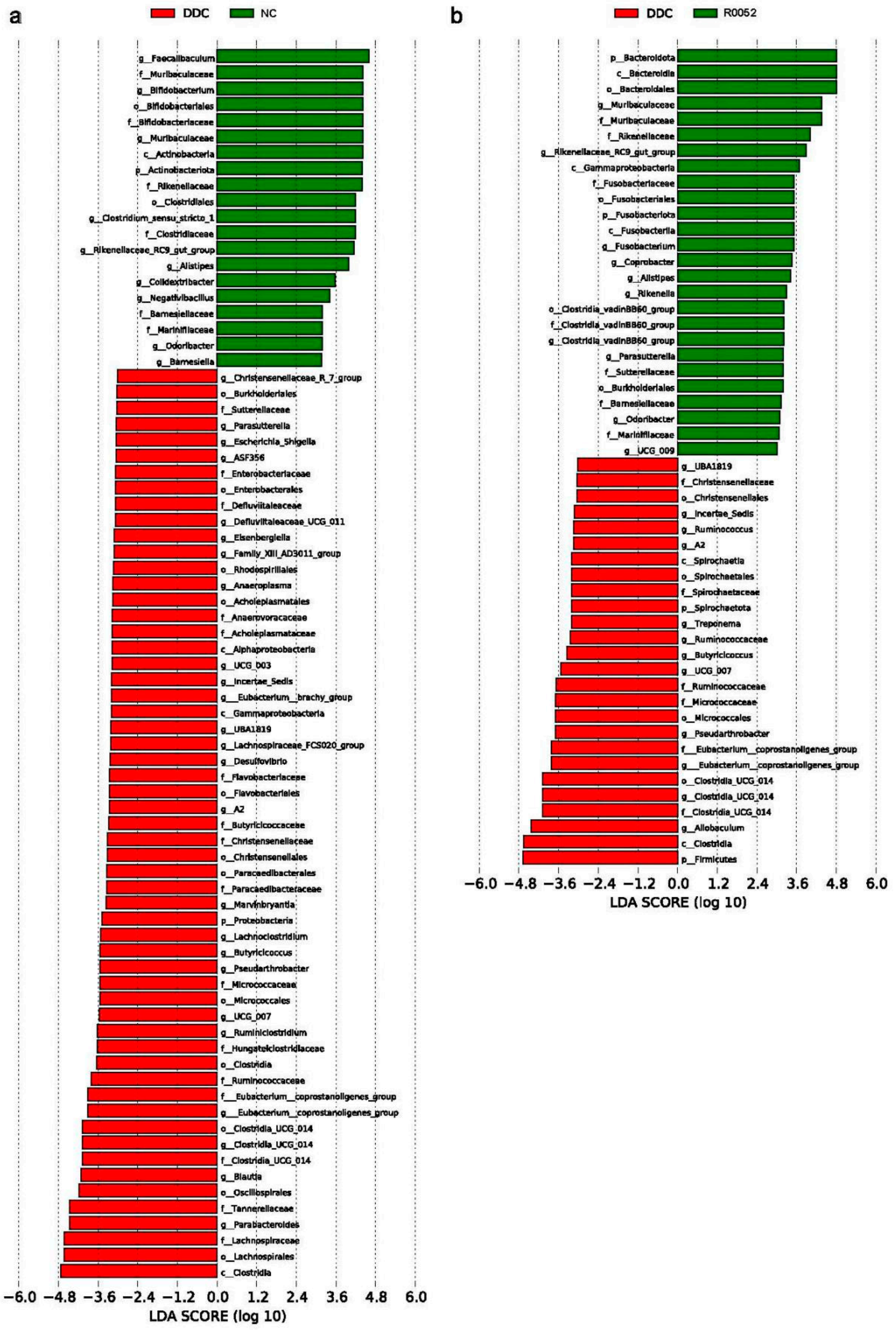

Figure S1. The LEfSe branching plot of gut microbiota in three groups mice. (a) LEfSe branching plot between the NC group and DDC group. (b) LEfSe branching plot between the DDC group and R0052 group.

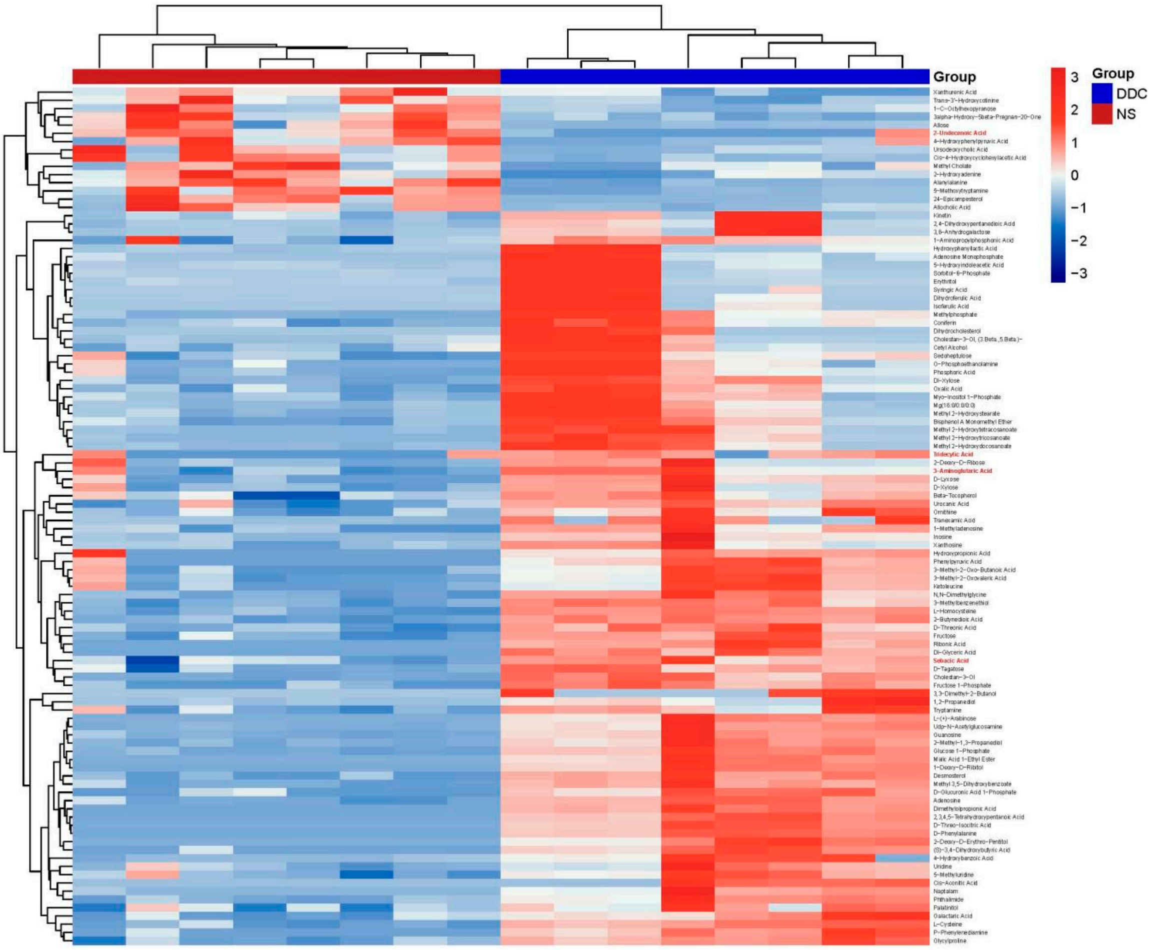

Figure S2. Heatmap of the different metabolites between the NC group and DDC group.

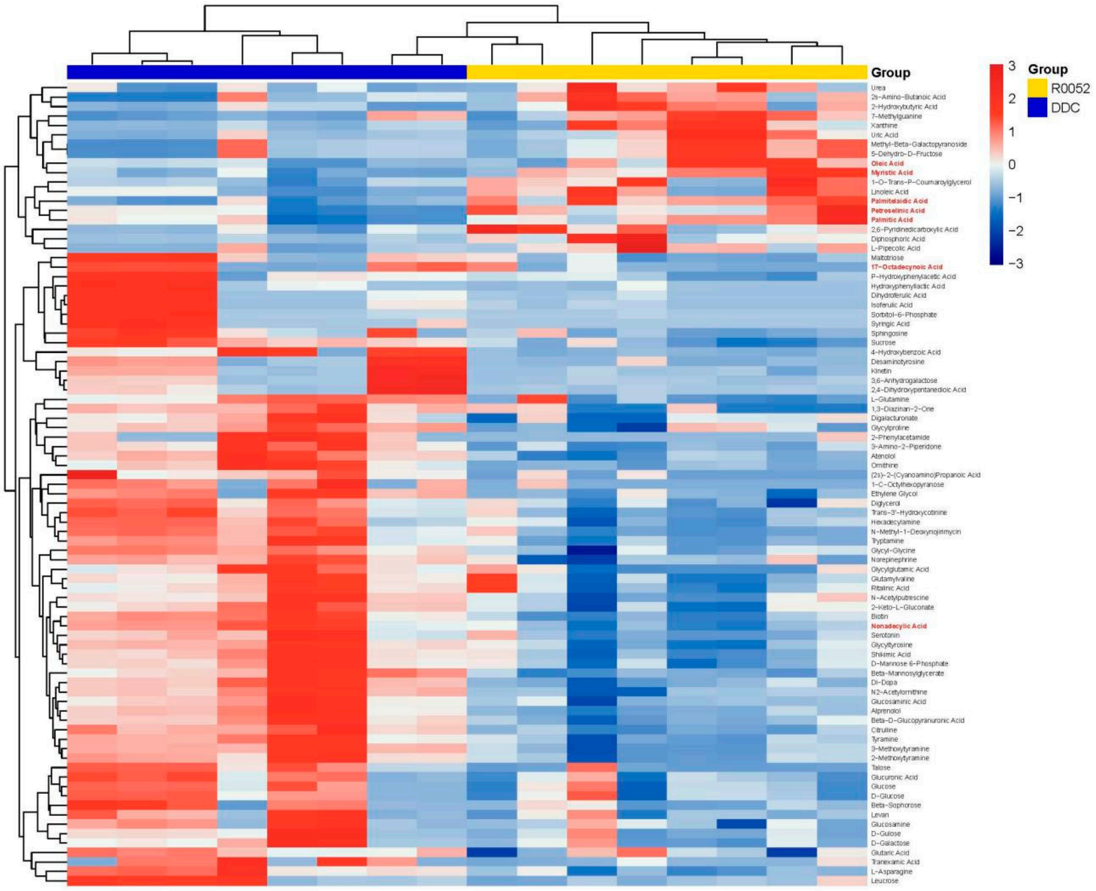

Figure S3. Heatmap of the different metabolites between the DDC group and R0052 group.

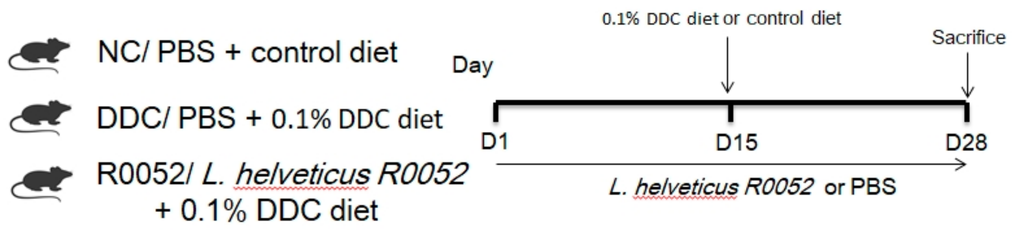

Figure S4. Experimental flowchart.

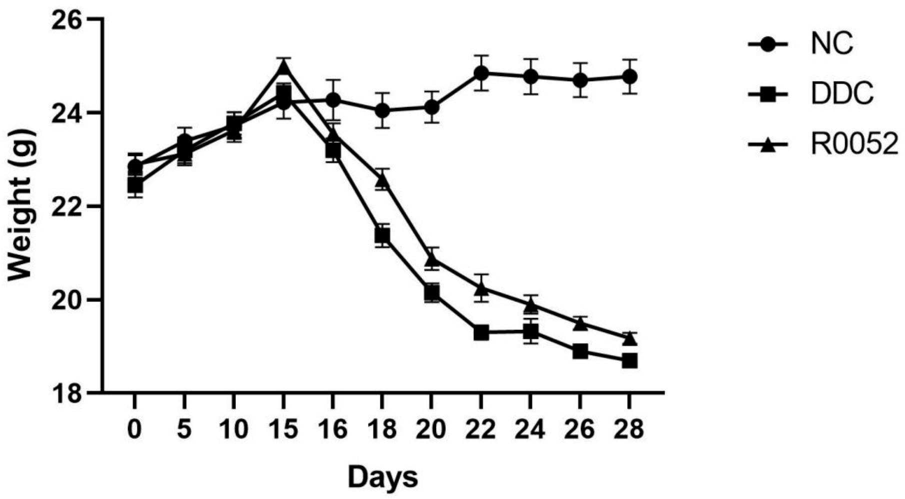

Figure S5. Daily mice body weight changes in NC, DDC and R0052 groups.

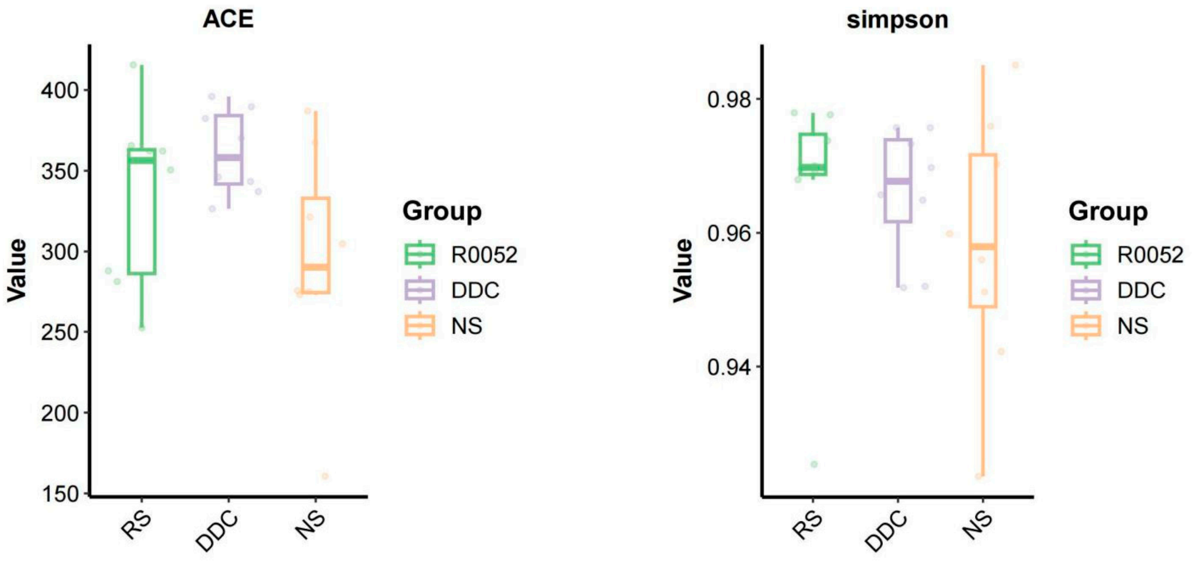

Figure S6. Alpha-diversity evaluation based on Chao1 and Simpson indexes.
